# Effective Design of Multifunctional Peptides by Combining Compatible Functions

**DOI:** 10.1371/journal.pcbi.1004786

**Published:** 2016-04-20

**Authors:** Christian Diener, Georgina Garza Ramos Martínez, Daniel Moreno Blas, David A. Castillo González, Gerardo Corzo, Susana Castro-Obregon, Gabriel Del Rio

**Affiliations:** 1 Department of Biochemistry and Structural Biology, Institute of Cellular Physiology, Universidad Nacional Autónoma de México, Mexico City, Mexico; 2 Department of Biochemistry, Facultad de Medicina, Universidad Nacional Autónoma de México, Mexico City, Mexico; 3 Department of Neurodevelopment and Physiology, Institute of Cellular Physiology, Universidad Nacional Autónoma de México, Mexico City, Mexico; 4 Department of Molecular Medicine and Bioprocesses, Institute of Biotechnology, Universidad Nacional Autónoma de México, Cuernavaca Morelos, México; University of California San Diego, UNITED STATES

## Abstract

Multifunctionality is a common trait of many natural proteins and peptides, yet the rules to generate such multifunctionality remain unclear. We propose that the rules defining some protein/peptide functions are compatible. To explore this hypothesis, we trained a computational method to predict cell-penetrating peptides at the sequence level and learned that antimicrobial peptides and DNA-binding proteins are compatible with the rules of our predictor. Based on this finding, we expected that designing peptides for CPP activity may render AMP and DNA-binding activities. To test this prediction, we designed peptides that embedded two independent functional domains (nuclear localization and yeast pheromone activity), linked by optimizing their composition to fit the rules characterizing cell-penetrating peptides. These peptides presented effective cell penetration, DNA-binding, pheromone and antimicrobial activities, thus confirming the effectiveness of our computational approach to design multifunctional peptides with potential therapeutic uses. Our computational implementation is available at http://bis.ifc.unam.mx/en/software/dcf.

## Author Summary

Most proteins and peptides in nature display multiple activities either by fusing different domains (with different activities) or by evolving multiple activities in a single domain. Understanding which activities may be combined to render multifunctional proteins remains an open question relevant to understanding the organization of living organisms and to improve the design of pharmacological peptides. To address this problem, we introduce the concept of compatible activities, that is, activities that may combine without losing any of these in a single polypeptide chain. To identify compatible activities in peptide sequences, we used a machine-learning approach and discovered that a penetrating activity should be compatible with DNA-binding and antimicrobial activities. To test if these activities may combine without any functional loss, we designed peptide sequences that harbor two independent activities (nuclear localization and pheromone) and experimentally showed that all our designed peptides display penetrability, pheromone, antimicrobial and DNA-binding activities, supporting the idea that multifunctionality may be achieved combining compatible activities.

## Introduction

The combination of multiple functions is an ubiquitous feature of naturally occurring proteins. More than 60% of the proteins in archaea and bacteria and more than 80% of eukaryotic proteins include more than one functional domain [1]. These numbers are further increased by a group of at least 200 moonlighting proteins, incorporating several functions in a single domain [2–4]. A similar situation has been described for peptides. For instance, some antibacterial peptides have the ability to either bind DNA, to penetrate cells or both [5,6]. Thus, multifunctionality in proteins and peptides seems to be a common feature in nature rather than an exception and it is then relevant to understand the basis for this diversity. In this sense, understanding how proteins acquire multiple functions is important to understand the structure-function relationship of proteins and aid in the design of polypharmacological peptides, an area of great interest in recent years to both academia and industry [7,8]. In the present work we report a novel computational method to design peptides with multiple functions.

Two kinds of relevant peptides with therapeutic application are cell-penetrating and antimicrobial peptides. Cell-penetrating peptides (CPPs) have the intrinsic ability to cross a variety of cellular membranes, which has been used for medical applications, particularly for cargo delivery [9–12]. Antimicrobial peptides (AMPs) on the other hand comprise a large class of naturally occurring peptides used to fight bacterial or fungal infections [13–15]. Fusing CPPs and AMPs has been shown to render multifunctional peptides useful for treating cancer, obesity and potentially many other diseases [16–19]. As such, it comes of no surprise that there have been ongoing efforts to design new cell penetrating or antimicrobial peptides [20–24]. However, fusing two or more activities into a peptide increases the peptide length and consequently its cost and immunogenicity, or may create an inactive peptide. Alternatively, the inclusion of several functions into a single antimicrobial peptide has medical potential as it allows to include mechanisms for specificity, organelle targeting or cargo delivery. As such it remains a challenge to design multifunctional antimicrobial peptides.

We have previously reported one computational strategy to create multifunctional peptides. Our designed peptides, referred to as Iztli peptides, embed the α-factor pheromone of *Saccharomyces cerevisiae* within an AMP sequence [25,26]. The characterization of these peptides leads us to propose that cell-penetrating and antimicrobial activities are closely related, that is, the design of an AMP may be compatible with a CPP activity and *vice versa*. We show here that this principle of compatibility can be used to design a set of multifunctional peptides, including two different functional domains and having an additional antimicrobial moonlighting activity. That is, we have designed CPPs with antimicrobial activity that embeds both pheromone and nuclear localization activities. This new design avoids some of the common limitations in the design of antibacterial peptides and produces multifunctional peptides. Our results may provide an alternative way to explore further the structure-function relationship of peptides and proteins.

## Results

### Identification of compatible domains and peptide design

The design of our peptides was based on two major assumptions. First, obtaining a probabilistic predictor for CPPs and optimizing peptide sequences to be consistent with this predictor may yield a compatible antimicrobial activity. Second, protein domains with different functions and high predicted CPP probability (or any other experimentally validated compatible function) should be particularly easy to embed in a peptide sequence that retains multifunctionality.

To this aim, we designed multifunctional peptides that would fit the rules for CPPs at the sequence level. We distinguished two different sets of CPPs: low and high efficient CPPs. Consequently, we tested several machine learning algorithms for their accuracy in identifying peptide sequences with CPP activity or efficient CPP activity. The accuracy (fraction of correctly predicted CPP sequences) was estimated from a 4-fold cross validation test (16 samples in total; the training set was randomly divided into 4 groups and in each turn, one group was retained as validation set and the rest was used for training). In agreement with previous results [27,28], we found that support vector machines (91% and 66% accuracies for CPPs and efficient CPPs, respectively) and random forests (90% and 67% accuracies for CPPs and efficient CPPs, respectively) yielded the best results. Here, random forests were much faster in training and rendering predictions and directly gave a probability estimate. Thus, here we used random forests for our study. The trained random forest predictor could now be used to assign a probability of being a CPP, P(CPP) or highly efficient CPP P(eff), to arbitrary peptide sequences.

**Fig 1:**
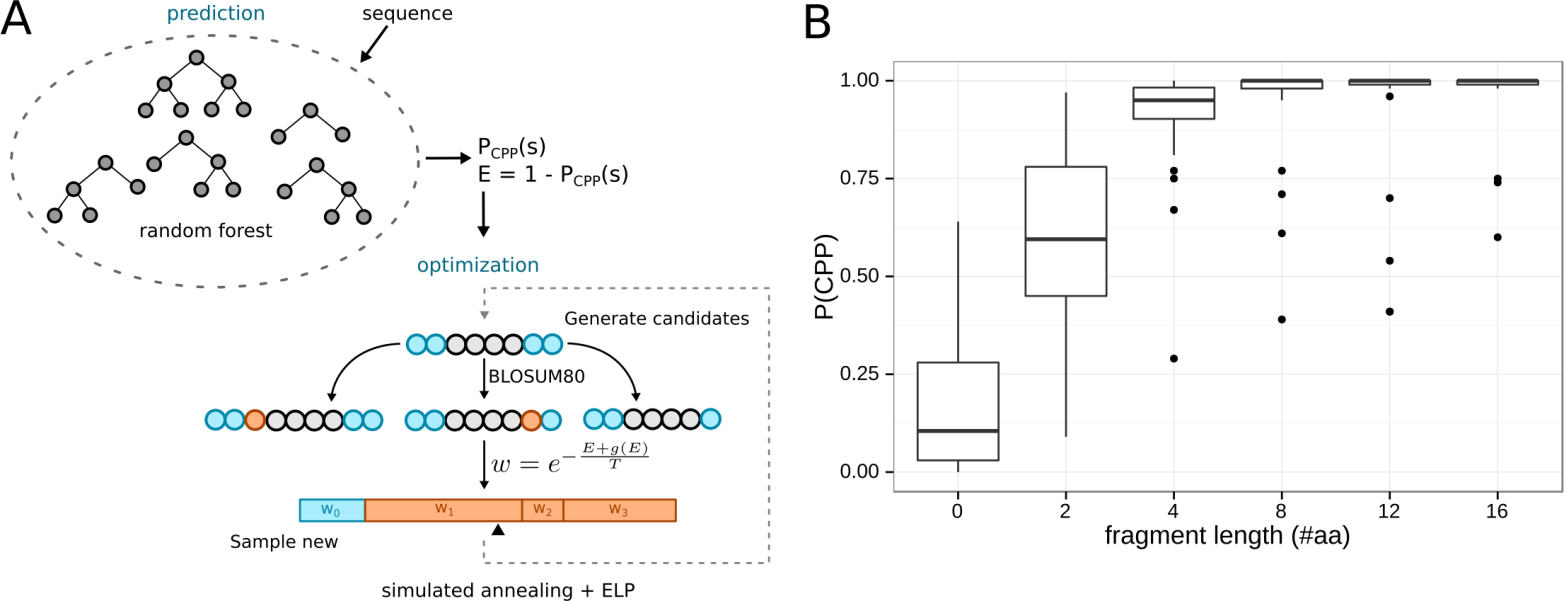
Identification and design of CPP-like sequences. (A) Methodology used to identify and design CPP-like sequences. First, a random forest predictor is trained on a set of known CPP and non-CPP sequences. The resulting probabilistic model is then used to identify CPP-like sequences and converted into an energy measure (E = 1 –P_CPP(S)_) for novel peptide design by simulated-annealing optimization (see Materials and Methods). (B) Algorithm performance during the design of CPP-like sequences. 32 random peptide sequences of length 16 were optimized with varying fragment lengths for 1000 iterations. Short fragments of more than 4 amino acids proved sufficient to transform non-CPP sequences to CPP-like sequences.

The described classification model was used to find compatible protein activities. According to our hypothesis, our model should predict peptide sequences with intrinsic AMP activity. To test this, we first predicted the mean probability to constitute a CPP and also be an efficient CPP, P(CPP, eff) = P(CPP)·P(eff), for all conserved domains sequences in the PFAM database (version 27.0, 14.831 protein families, >10 Mio. sequences, see Fig 1). This model yielded 82 protein families with P(CPP)>0.5 and only a single family, “Protamine P1”, with P(CPP, eff)>0.5 (for the complete distribution of P(CPP) values see S1 Fig and S1 Table). The 82 protein families were classified by their biological function and biological process GO terms as well as their descriptions in the PFAM database. Only 33 families had functional characterization in GO and 24 of these constituted DNA binding domains or structural components of the ribosome, thus, indicating that interaction with nucleic acids is compatible with CPP function. Families with antimicrobial activity formed the second largest functional group. 4 of the 9 families annotated as “antimicrobial” in PFAM were contained in the 82 CPP compatible families, as well as 3 additional antimicrobial families (Myotoxins, Ponericin and Mellitin) annotated in GO database. This was consistent with our aim and previous findings that CPP and antimicrobial peptides may be functionally related [26].

In summary, our results suggested that not only AMP but also DNA-binding activities are compatible with CPP rules. To validate these results, we designed several peptides that embed a Nuclear Localization Sequence (SV40 large T-antigen nuclear localization factor, NLS) and a ligand that is endocytosed (α-factor pheromone of *S*. *cerevisiae* [29]) within sequences that optimally matched CPP rules (see Table 1). Those optimal peptide sequences embedding other compatible activities were obtained by coupling the previously obtained random forest model with a Simulated Annealing optimization procedure. In this procedure, every peptide sequence with a compatible activity (here also referred to as peptide templates) were linked and flanked by short amino acid fragments (see Fig 1A) whose sequences are obtained from the optimization algorithm (see Materials and Methods).

**Table 1:** Peptides used in this study.

ID	Sequence	MW	P(CPP)	P(eff)	P(membind)
α-factor	WHWLQLKPGQPMY	1.68	0.39	0.5	0.34
NLS	QPKKKRKV	1.49	1	0.06	0.13
NLS-α	PKKKRKVWHWLQLKPGQPMY	2.55	0.89	0.45	0.08
NLS-CE	WRFVWMNPKKKRKV	1.90	0.97	0.75	0.09
**α-NLS-C**	RLWHWLQLKPGQPMYWRQPKSKRKVRR	3.56	0.91	0.59	0
**NLS-α-CE**	FRKWRRKPKKKRKVWWRKVKRRWHWLQLKPGQPMY	4.80	0.97	0.77	0.1
**chimera**	KRRWRFVWMNPKKKRKVPPWPYLLWWHWLQLKPGQPMY	5.01	0.88	0.63	0.16
MPG	GALFLGFLGAAGSTMGAWSQPKKKRKV	2.81	1	0.33	0.03
Iztli-1	KFLNRFWHWLQLKPGQPMY	2.49	0.64	0.41	0.22
TAMRA-α-NLS-C	TAMRA-RLWHWLQLKPGQPMYWRQPKSKRKVRR	3.97	ND	ND	ND
TAMRA-α-NLS-C woRK	TAMRA-ELWHWLQLEPGQPMYWEQPESEEEVEE	3.83	ND	ND	ND
TAMRA-NLS-α-CE	FRKWRRKPKKKRKVWWRKVKRRWHWLQLKPGQPMY	5.21	ND	ND	ND
TAMRA-NLS-α-CE woRK	FEEWEEEPEEEEEVWWEEVEEEWHWLQLEPGQPMY	5.03	ND	ND	ND
TAMRA-chimera	KRRWRFVWMNPKKKRKVPPWPYLLWWHWLQLKPGQPMY	5.01	ND	ND	ND
TAMRA-chimera woRK	EEEWEFVWMNPEEEEEVPPWPYLLWWHWLQLEPGQPMY	5.01	ND	ND	ND

The name of the peptide (column titled ID), the peptide sequence (column Sequence), the molecular weight in kDa (column MW), the calculated probability to match CPP rules (P(CPP)), the calculated probability to match efficient CPP rules (P(eff)) and the probability to match transmembrane protein regions (P(membind)) are indicated for each peptide tested in this study. Only NLS-CE, NLS-α-CE, and chimera were optimized for efficient CPP activity (in bold) and only chimera was also partially optimized for high membrane-binding activity. Peptides modified at the N-terminus with the fluorescent dye TAMRA (5-(6)-Carboxytetramethylrhodamine) are shown in this table as well. The peptides denoted with the “woRK” postfix (without RK) substitute every Arginine and Lysine residues in the corresponding peptide for Glutamic acids. ND stands for Not Determined.

This method was used to create three different peptides using three different designs. In our first design we placed α-factor before NLS and optimized only the probability to constitute a CPP-like sequence (named α-NLS-C). Here, three short fragments of two to three amino acids each were sufficient to obtain a P(CPP)>95%. In the remaining two designs, we switched the order of the templates and did not optimize the C-terminal end of the peptide since C-terminal modification of α-factor has been reported to inhibit its activity [30]. In the first design, the entire sequence was optimized to obtain a maximal joint probability of constituting a CPP-like sequence with high efficiency (named NLS-α-CE). In the second design, we generated a chimera peptide where the NLS template was flanked by a fragment constituting a high joint probability of being a CPP-like sequence with high CPP efficiency; the other fragment harboring the α-factor template was flanked by a fragment yielding a high joint probability of being a CPP-like sequence with similarity to membrane-binding sequences. As can be observed in Table 1, chimera peptide presents the largest P(membind) score in comparison with the other two designed peptides in this study (α-NLS-C and NLS-α-CE). The rules to bind membranes were included to test if these may improve CPP activity. This chimera peptide has a hydrophobic N-terminal NLS tail, a hydrophilic cationic C-terminal α-factor tail and a globally high probability of being CPP. During optimization we also chose the sequences with the largest predicted fraction of α-helices from the top 20 sequences. Table 1 shows the sequences of all designed peptides (listed in bold) and controls, together with their global predicted probabilities to match CPP rules (P(CPP)), or to match rules of highly efficient CPP (P(eff)) or a membrane-binding peptide rules (P(membind)).

### Multifunctionality of the designed peptides

**Fig 2:**
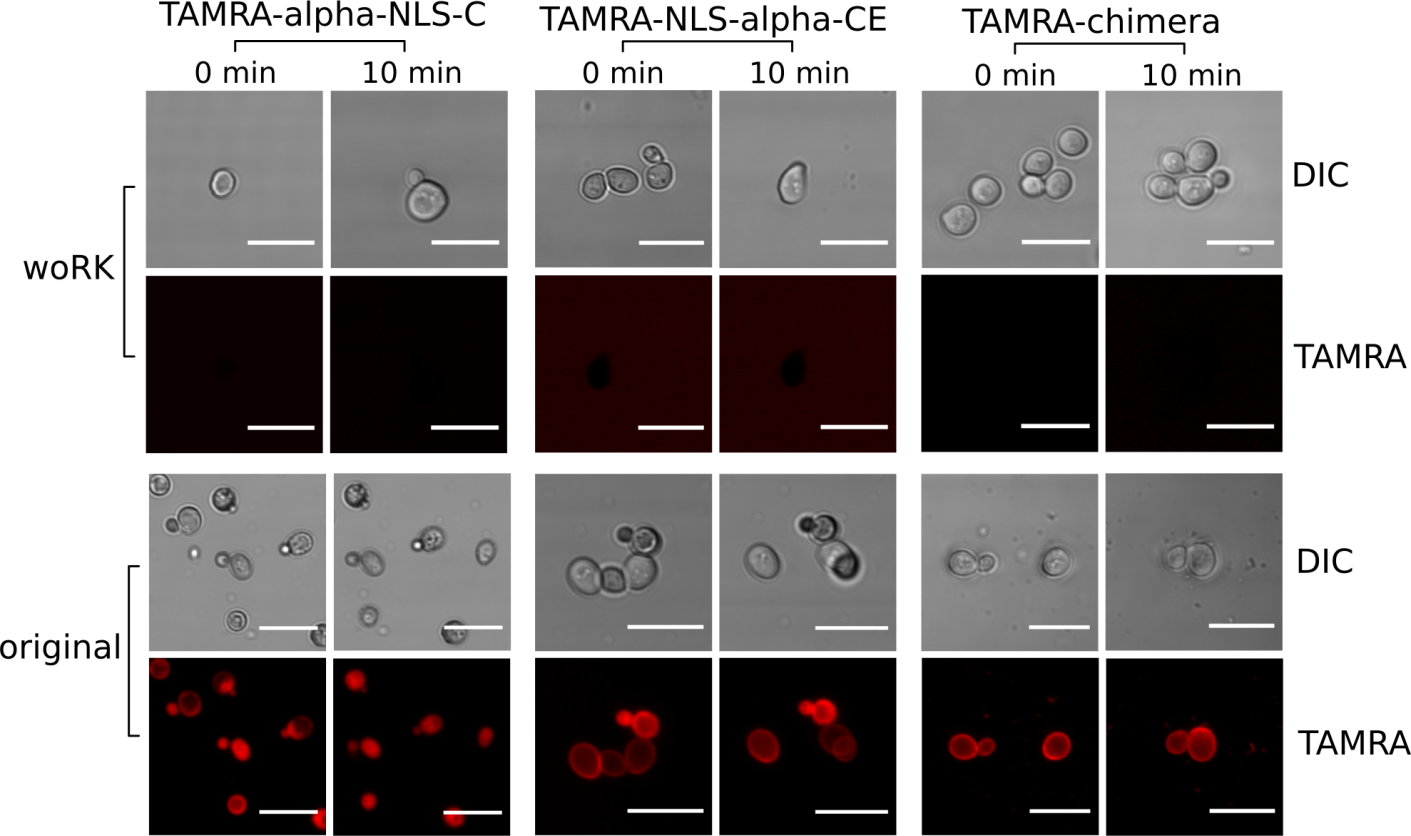
Designed peptides internalized into yeast cells. (A-F) Internalization assays in BY4741 yeast cells for peptide controls where Arg and Lys residues were replaced by Glu residues (A-C) and the original peptides (D-F). All peptides were used at 60 μM (final concentration) and labeled with the TAMRA fluorophore (see Table 1 and Materials and Methods for further details).

We first evaluated whether the designed peptides retained the activity of the templates. The presence of the NLS sequence may locate peptides in the nuclei of cells; for that to happen first the peptides have to be internalized. Hence, we tested the ability of these peptides to be internalized into two different cell types: yeast and mammalian cells. For yeast cells, confocal microscopy was used to evaluate CPP activity in our peptides (see Fig 2). Here, we used TAMRA-labeled versions of the peptides and as controls, variants of these peptides in which Lys and Arg residues were replaced by Glu residues (woRK peptides in Fig 2). α-NLS-C, NLS-α-CE and chimera did all quickly accumulate in the cell membrane and inside the yeast cells, whereas none of the controls were detected inside the yeast cells within the first 10 min.

**Fig 3:**
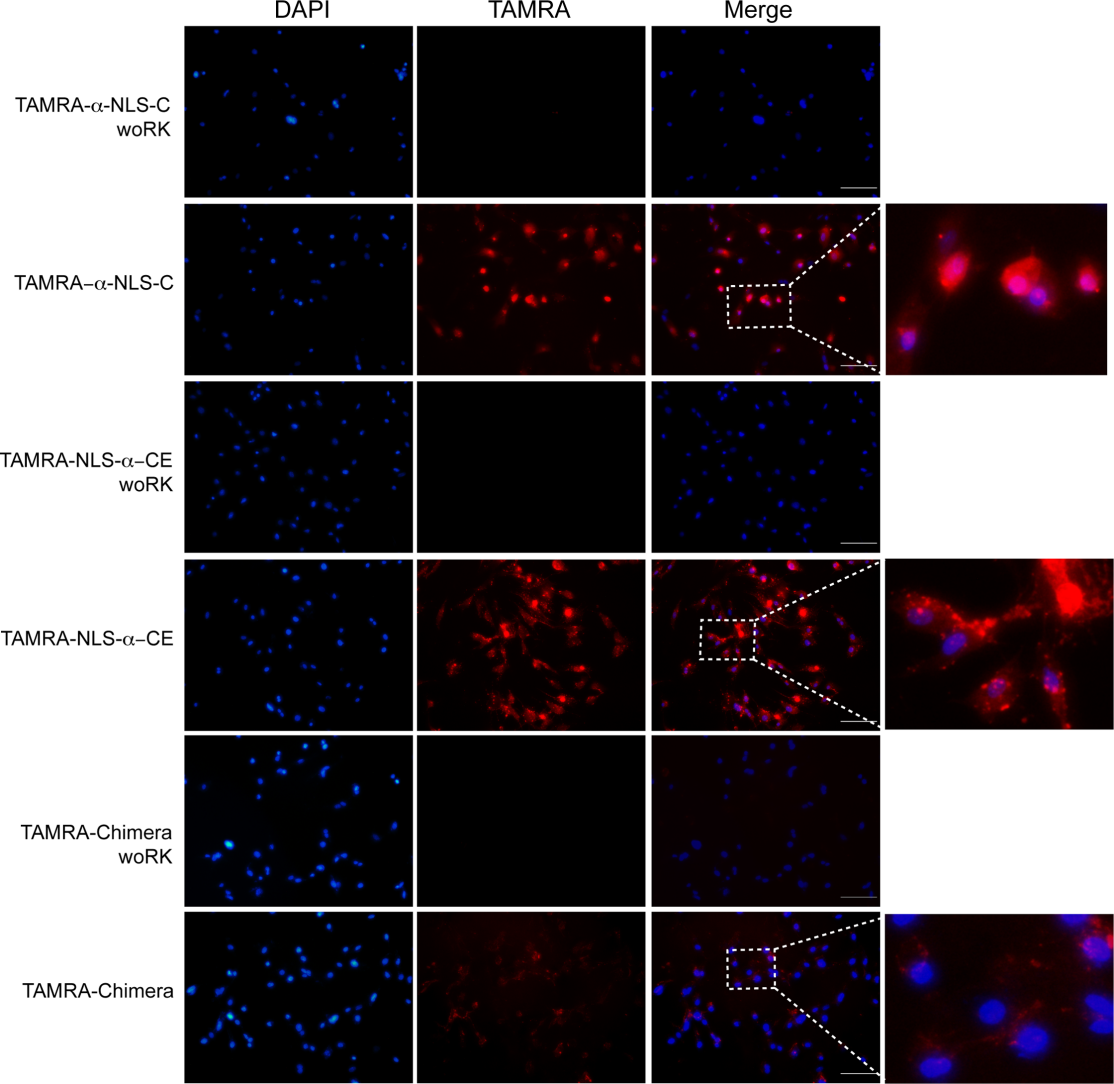
Designed peptides internalized and reach nuclear localization in mouse cells. Primary cultured MEF cells were exposed for 20 min to 3 designed CPPs labeled with TAMRA at the N-terminus (TAMRA-α-NLS-C, TAMRA-NLS-α-CE and TAMRA chimera; see Table 1) or their respective control peptides (TAMRA-α-NLS-C woRK, TAMRA-NLS-α-CE woRK and TAMRA-chimera woRK; see Table 1). All peptides were used at a final concentration of 60 μM. To evaluate the co-localization of these peptides with nuclear DNA, cells were stained with DAPI after 20 min. The figure shows the fluorescence observed in DAPI, TAMRA and merged images to show the co-localization of DAPI and TAMRA signals; the squares indicate the area magnified to the right. The scale bar represents 100 μm.

Next, we tested the ability of these peptides to be localized also in mammalian cells, and whether they would reach the nuclei. Here, we performed internalization assays in mouse embryonic fibroblasts (primary cultures) in which nuclear DNA was stained with DAPI after 20 min of cells being exposed to the designed peptides or the corresponding “woRK” controls (see Methods). By fluorescence microscopy we observed indeed both internalization and nuclear localization (co-localization with DAPI) only for the designed peptides, but not for the control “woRK” peptides (see Fig 3).

**Fig 4:**
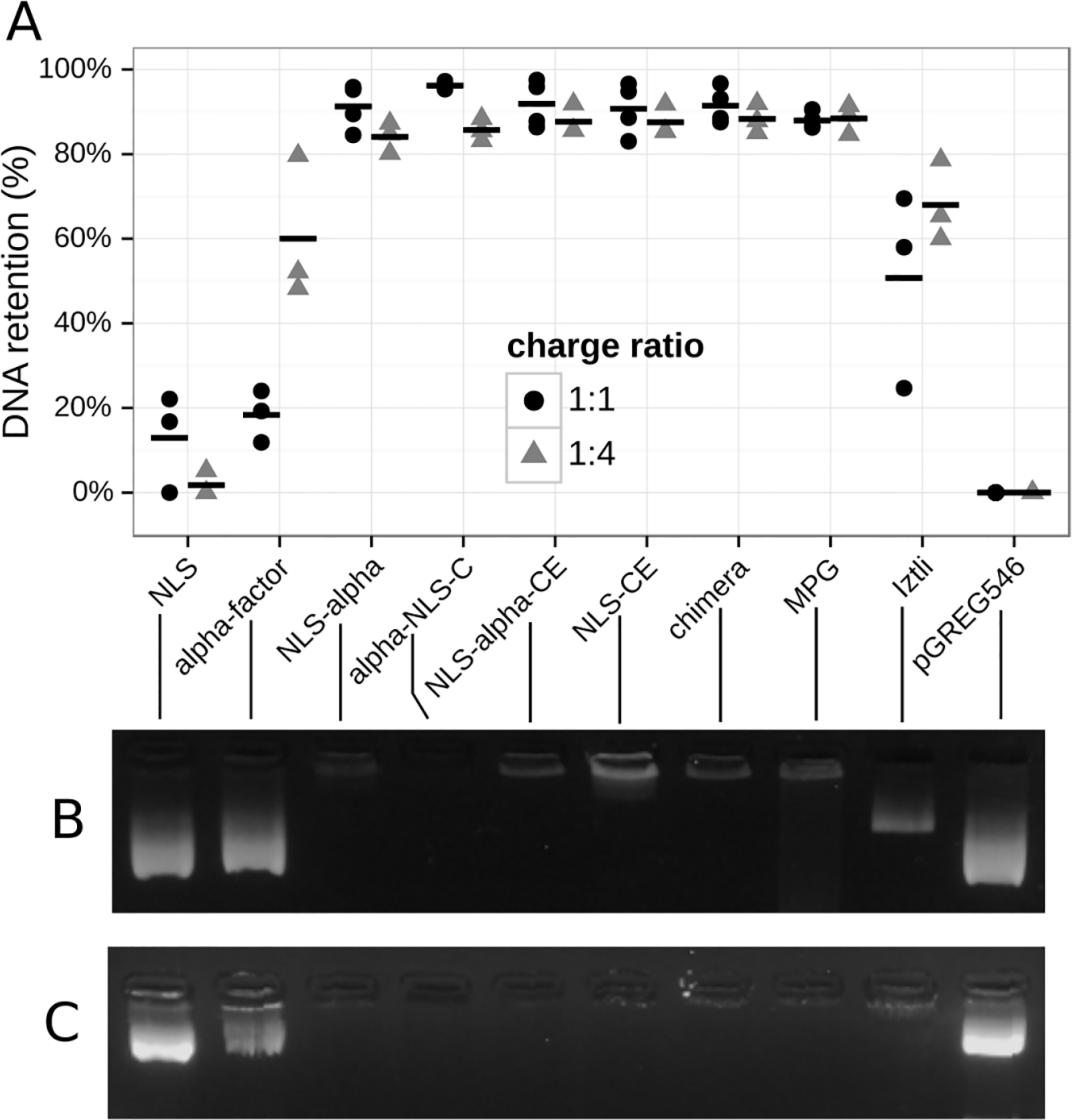
DNA binding. (A) Quantification of DNA retention obtained from three independent electrophoretic mobility shift assays. All measurements are shown and horizontal bars denote the sample mean. Representative gels are shown in (B-C).

The co-localization of TAMRA (labeled peptides) and DAPI (nuclei) fluorescence indicated that the designed peptides are also internalized into MEF cells and localize at the nuclei, possibly interacting with DNA. To test the ability of the designed peptides to bind nucleic acids, we evaluated by electrophoretic mobility shift assays (EMSAs) the retention of DNA by the designed peptides. Each EMSA was executed with peptide quantities that were multiples of the DNA to peptide charge ratio. A DNA to peptide charge ratio of 1:1 is represented by the number of positive charges of the peptide required to neutralize the negative charges of the DNA backbone. Retention in the gel is an indirect measure of the fraction of positive charges within the peptide participating in binding the DNA molecules. All EMSA assays were performed using the yeast plasmid pGREG546. As negative control, pGREG546 without any peptide was used, and as positive control the known DNA-binding cell penetrating peptide MPG was used [31,32]. We observed that NLS alone was not capable of binding DNA in low concentrations. However, retention in the gel was observed when NLS was joined to any larger peptide fragment. Furthermore, all designs including the NLS sequence showed almost complete DNA retention at the minimal DNA to peptide charge ratio of 1:1 and this behavior remained the same when increasing the peptide concentrations to yield a DNA to peptide charge ratio of 1:4 (see Fig 4A–4C). α-factor alone showed a weak ability to bind DNA. DNA retention was around 60% in the 1:4 DNA to peptide charge ratio suggesting that only a small fraction of the positive charges in α-factor participate in DNA-binding. Adding positive charges to the N-terminus of α-factor increased its affinity for DNA, such as in the case of the Iztli peptide 1 [25]. These results indicate that increasing the number of positive charges in peptides, as it often occurs in CPP design, promotes the peptides' affinity for DNA and supports that these activities are compatible. Nevertheless, DNA binding may be governed by diverse structural factors, but our results show that positively charged residues contribute to it. It should be noted that the optimized peptides showed the same *in vitro* DNA affinity as MPG, a peptide known to bind DNA *in vivo* [24].

**Fig 5:**
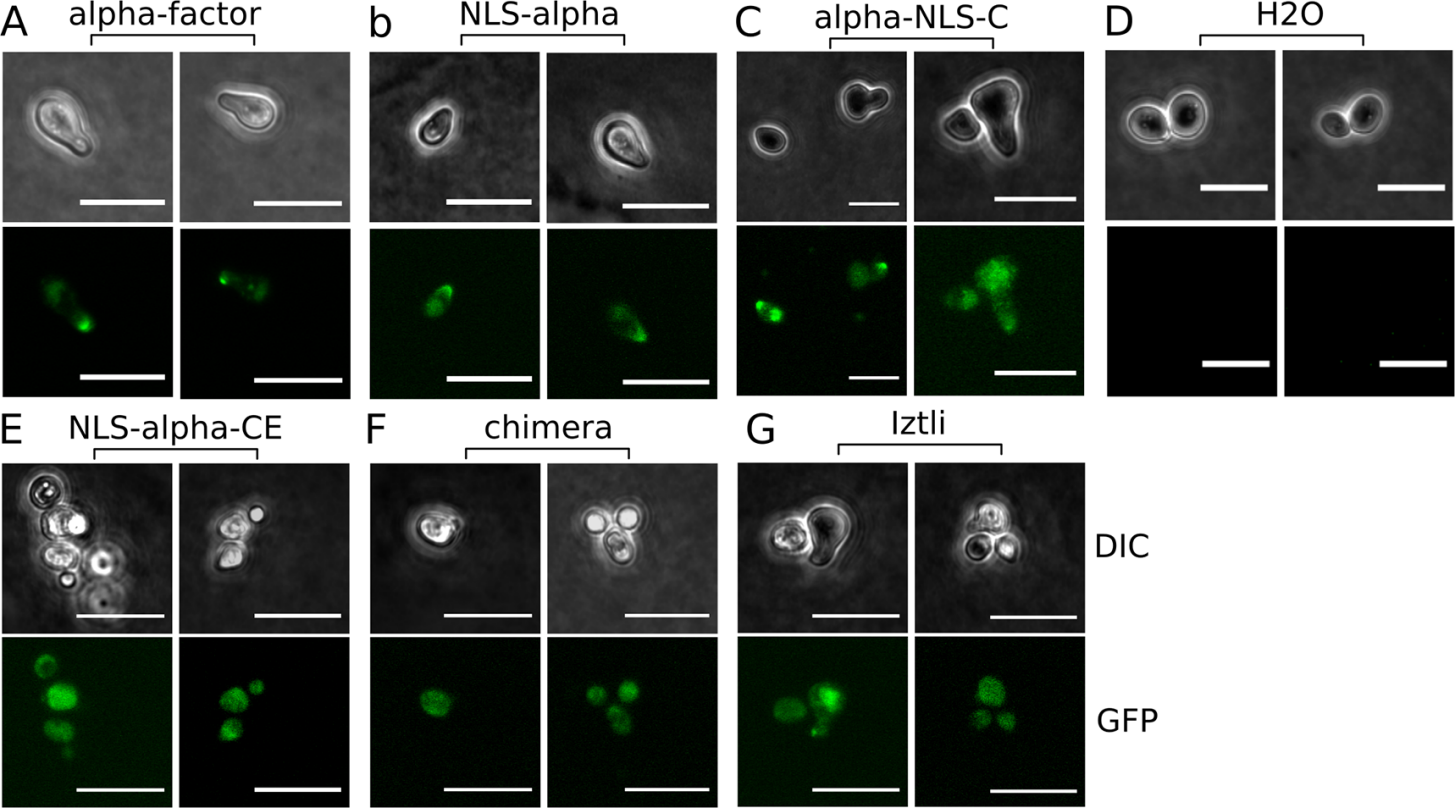
Pheromone activity. The pheromone signaling pathway is confirmed by the expression of Fus1-GFP, characteristic “shmoo” phenotype and the absence of budding observed in differential interference contrast (DIC) bright field. Concentrations were 20 μM for α-factor and 60 μM for all other peptides. The negative control was water without any peptide.

Finally, the activity of the α-factor embedded in the peptides was evaluated by activation of the pheromone-signaling pathway in *MAT*a cells of *S*. *cerevisiae*. We monitored the pheromone signaling activity by following the expression of the GFP-labeled Fus1 protein. Fus1 is among the proteins with the highest pheromone-induced expression and is required for cell-cell fusion during mating [33,34]. NLS-α and α-NLS-C showed significant induction of expression of Fus1-GFP, however, higher concentration of both peptides is required compared to the free α-factor (see Fig 5A–5G). The observed phenotype was consistent with the activation of signaling showing a mating protrusion, often called “shmoo” as well as the absence of budding due to inhibition of the Cdc28 cyclin [35,36]. NLS-α-CE and chimera showed partial shmoo-like phenotypes, a diffuse GFP, strong vacuolar fragmentation and a less refractive membrane in differential interference contrast bright field. As the same phenotype could also be observed for Iztli-1, a known fungicide, it is likely that this phenotype was the consequence of cellular damage.

In conclusion, optimizing the designed peptides by our algorithm enabled the DNA-binding activity of the NLS subunit both *in vitro* and *in vivo*, presented the CPP activity and retained the pheromone activity of the α-factor subunit, although this last activity was reduced probably caused by cellular damage.

### Antibacterial activity

**Fig 6:**
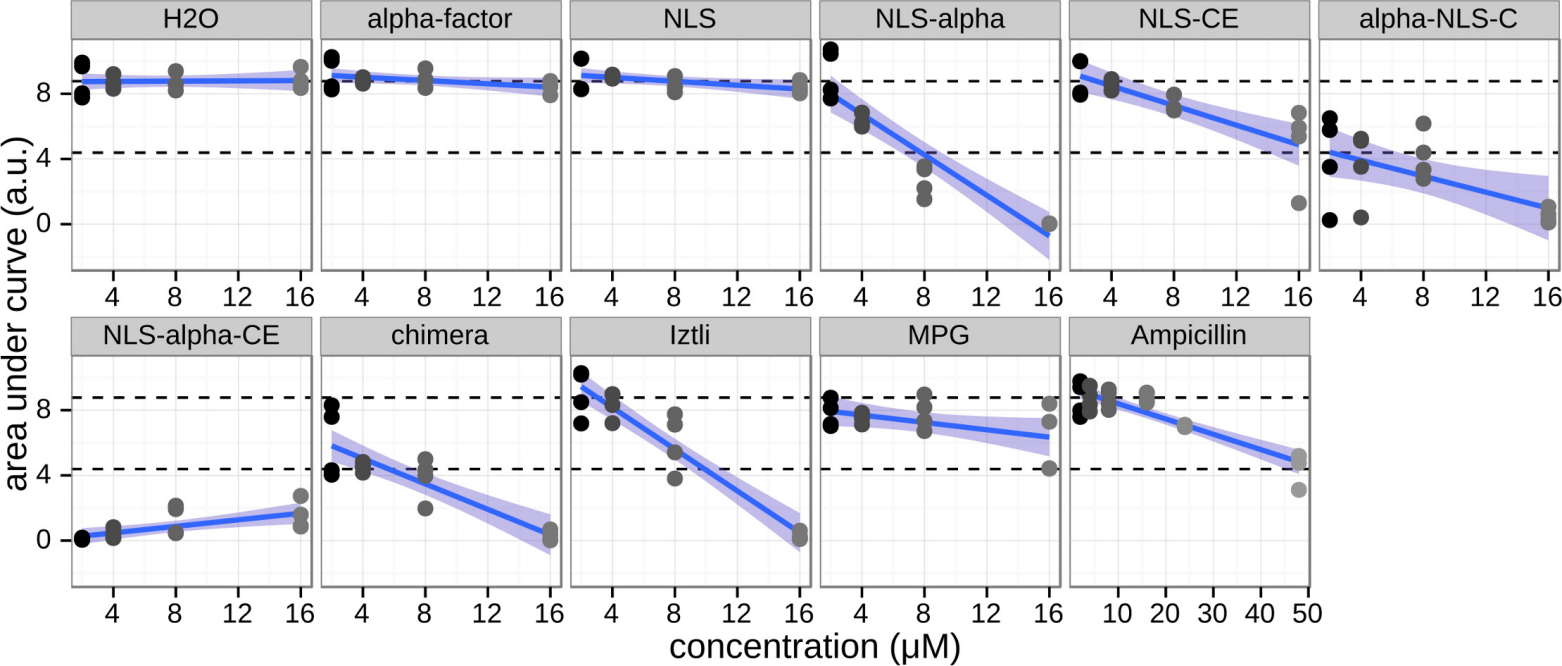
Antibacterial activity. DH5α *E*. *coli* cells were treated with varying concentrations of peptides ranging from 0 to 16 μM for the peptides and 0 to 48 μM for Ampicillin. The relative area under the growth curve measured the growth inhibition. Measurements from four repetitions distributed onto two different plates are shown as dots colored by their respective concentrations. Linear regressions are shown as blue lines and their respective standard errors as blue shades. Dashed lines indicate the mean area under the curve for the H2O controls and its 50% value respectively. Minimal inhibitory concentrations to achieve 50% or 100% of cell growth inhibition are reported.

If CPP, DNA binding and AMP activities are compatible, the designed peptides should inhibit bacterial growth. Here we exposed *E*. *coli* DH5α cells to varying low concentrations of the designed peptides as well as several controls (see Fig 4). All designed peptides inhibit *E*. *coli* growth, with NLS-α-CE being the most potent inhibitor abolishing *E*. *coli* growth completely at concentrations as low as 2 μM. This peptide also induced flocculation of *E*. *coli* cultures, as noted by the higher initial OD (see S2 Fig). Both α-NLS-C and the chimera peptide showed partial growth inhibition at 2 μM and 50% growth inhibition at 16 μM. However, α-NLS-C activity showed large variation, particularly between different plates, an indication of high sensibility to initial conditions (see S2 Fig). Surprisingly, coupling the NLS sequence to α-factor without optimization was sufficient to yield an antibacterial activity providing partial growth inhibition at 4 μM and 50% inhibition at 16 μM. To test whether α-factor was important for AMP activity we also tested pure α-factor, pure NLS, the optimized NLS fitting CPP rules (NLS-CE) and the Iztli-1 peptide, a previously reported peptide with an embedded α-factor specifically designed to have antibacterial activity [25]. We observed that pure α-factor and NLS had no antibacterial activity, whereas NLS-CE yielded a partial growth inhibition of *E*. *coli*. The Iztli-1 peptide showed a very similar *E*. *coli* growth inhibition as NLS-α, probably due to the slightly positive charge of its N-terminal tail, resembling NLS. This indicates that the antibacterial activity observed in NLS-α-CE, α-NLS-C and the chimera peptide is a synergistic effect between the aggregation of NLS, α-factor and the CPP optimization. A linear trend could be observed between concentration and growth inhibition (regression lines with standard deviations shown in Fig 6). We used this linear adjustment to predict and test the required concentrations to completely inhibit growth in *E*. *coli*. The minimal inhibitory concentrations for *E*. *coli* are reported in Table 2 (growth curves are shown in S4 Fig). It is noteworthy to observe that the NLS-α-CE peptide is a more potent antibiotic than Ampicillin.

**Table 2:** Minimal inhibitory concentrations for the tested peptides.

ID	*E*. *coli* (MIC for 50% inhibition, μM)	*E*. *coli* (MIC for 100% inhibition, μM)
α-NLS-C	8	20
NLS-α-CE	<2	2
Chimera	4	22
Iztli-1	8	20
Ampicillin	>48	100

Concentrations of peptides to achieve 50% of growth inhibition (MIC for 50% inhibition) or 100% of growth inhibition (MIC for 100% inhibition) for *E*. *coli*.

### Peptide secondary structure contents

**Fig 7:**
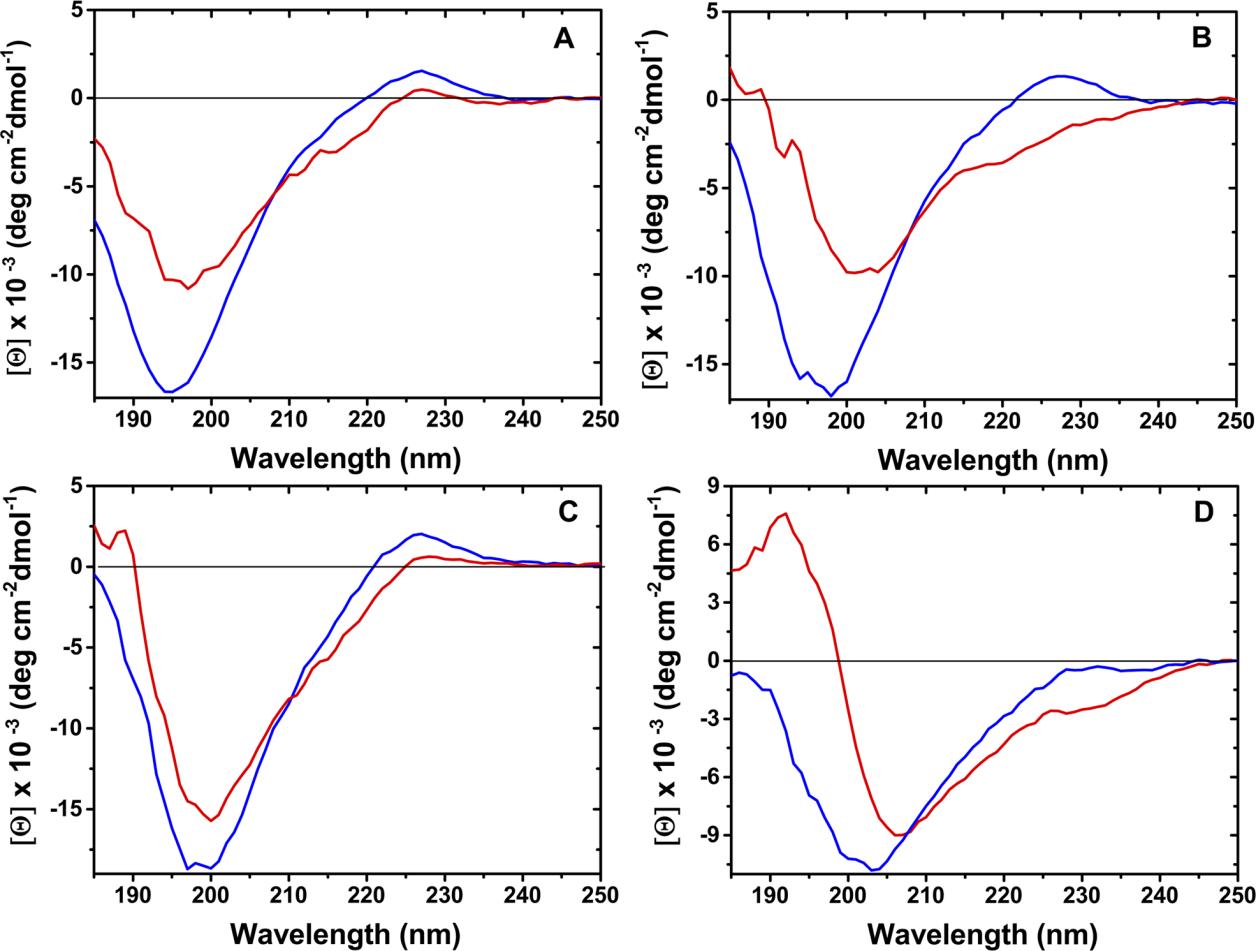
CD spectra of peptides. Spectra of peptides in water (blue line) and 50% TFE (red line) for NLS-α (A), NLS-α-CE (B), NLS-CE (C) and chimera (D) are presented.

We studied the structural properties of the peptides *in vitro*, using circular dichroism (see Materials and Methods). The far-UV CD spectra for NLS-CE, NLS-α and NLS-α-CE in water matched the characteristics of a random coil conformation. These spectra had a minimum of 195 nm for NLS-α, 197 nm for NLS-α-CE and 199 nm for NLS-CE (Fig 7A–7C). The presence of tryptophan in these peptides resulted in a positive band between 220 to 235 nm. Similar results were observed in 50% TFE, which confirmed the unstructured nature of these peptides in this solvent. The CD spectra of NLS-α and NLS-α-CE peptides with 50% TFE displayed a decrease in negative ellipticity, suggesting that the TFE promoted aggregation of the peptides (Fig 7A and 7B). The analysis of secondary structure content showed nearly identical fractions of α-helices, β-sheets and random-coil of peptides in water and in 50% TFE (Table 3). Interestingly, the CONTIN/LL estimates 30 to 35% content of β-sheets regardless of the solvent.

**Table 3:** Proportion of secondary structures observed in the designed peptides.

Peptide	Solvent	Secondary Structure (%)		
		α-Helix	β-Sheet	Random coil
NLS-α	Water	5	28	67
	TFE	6	35	59
NLS-α-CE	Water	6	30	64
	TFE	10	31	59
NLS-CE	Water	6	31	64
	TFE	5	34	61
Chimera	water	8	33	59
	TFE	24	28	48

The CD spectrum of the chimera peptide in water is characterized by a strong minimum shifted to 204 nm. A conformational change to a more ordered structure could be induced in the presence of 50% TFE. The positive maximum at 192 nm and a negative minimum at 207 nm together with a weak increase of negative ellipticity around 222 nm, suggested the presence of a mixture of α-helix and β-sheet in equilibrium with random coil conformation. Deconvolution of the CD spectrum of chimera in 50% TFE indicated some gain in α-helical content (from 8 to 24%), with the concomitant decrease in both the β-sheet and random-coil contents (Table 3). Thus, none of the peptides showed a strong tendency towards a particular structure in water, and only the chimera peptide showed a slight tendency towards an α-helical structure in TFE.

## Discussion

We have presented a new strategy to design multifunctional peptides. Three multifunctional peptides were designed, chemically synthesized and structurally and biologically tested. The basic idea of our method is that proteins share particular activities (*e*.*g*. CPP, AMP and DNA binding) that are compatible, and consequently they can be placed together in multifunctional peptide sequences. This functional compatibility can be predicted at the protein sequence level by training a probabilistic classifier. In this work, we trained a classifier with CPPs and non-CPPs and discovered that both DNA-binding and AMP activities are compatible with CPP rules (see S1 Fig and S1 Table). To validate this prediction, we tested if CPP rules could be used to design peptide sequences with AMP and DNA-binding activities and we showed that it is possible to do so. An important aspect of our designs is that two additional activities were incorporated (pheromone and nuclear localization) within these multifunctional peptides and we showed that these additional activities were retained, indicating that compatible functions enable multifunctional and moonlight polypeptides. As such, our approach may extend previous computational methods that effectively predict peptides with CPP activity [22–24] to effectively design multifunctional peptides [20–21].

Here, it became apparent that it is possible to gain antibacterial activity when mimicking CPPs. Interestingly, the antibacterial activity could be gained with the optimization of a single domain (as shown by NLS-CE) but was much stronger when including the α-factor sequence into the entire peptide sequence. As demonstrated by Iztli-1 and NLS-α, α-factor seems to have an intrinsic ability to be transformed into an antibacterial peptide by adding positive residues to it. For instance, embedding the α-factor within an AMP domain maintained the pheromone activity (see Fig 5) and previous results showed that this is true for concentrations as low as 10μM [25,26]. Embedding the α-factor in any other multifunctional peptide optimized for CPP also maintained the pheromone activity (see Fig 5B, 5C, 5E and 5F), yet at higher concentrations. This observation was true independent of the location (N or C terminus) of the extra residues added to the α-factor. These results suggest that the structure of the α-factor pheromone matches better the AMP rules than the CPP rules. Considering the known function of the α-factor to partially induce cell death on yeast cells, it may indeed be considered that the α-factor is more related to AMP activity than to CPP activity and consequently, its presence within multifunctional peptides optimized for CPP rules may explain the gain in AMP activity [37,38]. Additionally, any peptide sequence optimized to fit CPP rules (NLS-CE, α-NLS-C, NLS-α-CE and chimera) also showed effective DNA-binding activity (see Fig 4A–4C). Thus, peptides optimized for CPP rules are compatible with DNA-binding activity, as expected from our analysis on the PFAM database (see S1 Fig and S1 Table). This makes a strong argument for multifunctional designs of antimicrobial peptides since it is not only possible to do so, but the antibacterial activity can be amplified by an order of magnitude when including a specific set of compatible domains.

In terms of secondary structure, the α-helical structure is frequently found among AMPs as well as in DNA-binding domains [39,40]. An important difference is that for AMPs the helical region participates directly in the biological activity while for DNA-binding, the binding may or may not take place at the helix. None of our designed peptides presented a strong preference to adopt an α-helical structure neither in water nor in TFE (see Table 3 and Fig 7), suggesting that the functionality of the designed peptides does not depend on this particular structure. Instead these results suggest that the peptides we designed posses some conformational freedom characteristic of multifunctional proteins [41]. It is not the goal of this work to establish the structural basis of the activity of these peptides, and the secondary structure of these peptides in complex with DNA or membranes remains to be elucidated. In any case, our results provide additional evidence for the conformational freedom of multifunctional peptides.

Comparing the efficiencies to penetrate cells, to kill *E*. *coli* cells or to bind DNA among the three designed peptides (α-NLS-C, NLS-α-CE and chimera), it may be observed that each peptide perform similarly in most of these activities, but some differences were noticed. For instance, NLS-α-CE was clearly the best antibacterial peptide, but had similar penetrating activity in yeast or mammalian cells than α-NLS-C. On the other hand, chimera had the lowest antibacterial and penetrating activities. Concerning the design of these peptides, NLS-α-CE sequence was optimized to match efficient CPP rules, while α-NLS-C was optimized to match CPP rules, without considering their efficiency. On the other hand, the chimera peptide included optimization to match membrane-binding proteins. Thus, these results suggest that the optimization to match efficient CPPs rendered highly efficient CPP and AMP activities, while optimization for membrane binding proteins reduced these activities. Yet, other aspects of these peptides varied as well (*e*.*g*., the length of the peptides), thus further studies are required to explain this observation.

Our results pose two interesting questions. From an evolutionary perspective, compatible functions may facilitate a change of function or moonlighting activity. As such, there might be an evolutionary connection between compatible functions and multifunctional proteins, and such a relationship deserves to be further investigated. From a pharmacological perspective, it has been recognized that a shift from the one-target drug model to a multi-target paradigm is required, considering that drugs with clinical effects often have multiple targets [42]. The method we developed here might facilitate the design of poly-pharmacological peptides. Thus, it will be useful to investigate further the impact of creating this type of multifunctional peptides for treating complex diseases.

In summary, we introduce a conceptual framework to design multifunctional CPPs based on the observed compatibility of certain peptide activities. We also present a design strategy and experimental evidence for three new multifunctional peptides that combine cell penetration, antimicrobial, pheromone and DNA binding activities. Our software is available at http://bis.ifc.unam.mx/en/software/dcf.

## Materials and Methods

### Strains and peptides

The bacterial strain used in this study was *Escherichia coli* DH5α((F- endA1 glnV44 thi-1 recA1 relA1 gyrA96 deoR nupG ϕ80d*lacZ*ΔM15Δ(*lacZYA-argF*)U169, hsdR17(rK- mK+), λ–)). For the pheromone signaling we used the *Saccharomyces cerevisiae* BY4741 strain (*MAT*a his3Δ1 leu2Δ0 met15Δ0 ura3Δ0).

The mating pheromone α-factor from *S*. *cerevisiae* was purchased from Sigma-Aldrich (catalog number T6901). Anaspec, Inc. produced the Iztli peptide 1 (Iztli-1). The company verified the purity of these peptides using High-performance liquid chromatography (HPLC) and mass spectrometry (data not shown). Optimized Fmoc and Boc methodologies were employed for peptide syntheses with free N- and C- termini. Delivered peptide powders were first dissolved in sterile ddH_2_O and their actual concentrations were determined at 280 nm using a Nanodrop apparatus (Thermo Scientific, USA). A small fraction was subsequently diluted further to yield 200μM stock solutions kept at -80°C. All peptides used in this study included only L-amino acids. AnaSpec Inc also synthesized the TAMRA-labeled versions of the peptides used in this study. We purified these peptides using HPLC and verified their masses by mass spectrometry. The purity of these peptides was estimated as follows: 1) α-NLS-C: >95%, 2) α-NLS-C woRK >95%, 3) chimera >80%, 4) chimera woRK >50%, 5) NLS-α-CE >80% and 6) NLS-α-CE woRK >80%.

### CPP prediction

Classification was performed on a compound data set of 1267 confirmed CPP sequences and 1267 random peptides from SWISS-PROT using the classification algorithms from alglib and dlib (www.alglib.net, www.dlib.net). For CPP and CPP efficiency classification we used data sets obtained from http://www.imtech.res.in/raghava/cellppd/dataset.php. In particular, the data set for CPPs consisted of the CPPSite1, Sanders and Dobchev sets [43]. The membrane-binding data set was obtained from a previously published list of trans-membrane protein fragments [44]. All training sets are also available in the Github repository at https://github.com/cdiener/dcf. For each of the sequences 27 physiochemical properties were calculated, consisting of the frequencies of each of the 20 amino acids and 7 additional properties. All of those additional properties were calculated across a sliding window of 8 amino acids and summarized by either the mean or the difference of maximum and minimum values. The additional properties were: mean charge, sliding window range of charge, hydrophobicity, isoelectric point, sliding window range of the hydrophobic moment, water-octanol partition coefficient and an approximation of the alpha-helical content in the sequence. Those properties were then used as the features for random forest and SVM classification. Probabilities for any new sequence to constitute a CPP could now be predicted from the trained models. For prediction of CPP efficiency and membrane-binding the models were trained in the same manner using data sets of highly efficient versus low efficiency CPPs and membrane-binding versus non membrane-binding domains, respectively.

PFAM domains were analyzed by first obtaining version 27.0 of the PFAM database containing only the sequence parts used in family identification (conserved regions). This data set was then split into the individual families and the CPP probabilities P(CPP) = P(CPP|s) were predicted for all individual sequences in the data set. Family-wise measures were obtained by calculating the mean probabilities to be a CPP, P(CPP) and mean probability to be a CPP and have high efficiency P(CPP, eff) = P(CPP, eff|s) for each family. Source code and all data for these classifications, can be found at http://bis.ifc.unam.mx/software/dcf (DOI: 10.5281/zenodo.30278).

### Automated peptide design

Optimization of peptide fragments joining and flanking the desired domains was performed by a custom implementation of a Simulated Annealing algorithm in C++ called modes (Multi-objective designer). New candidate sequences were generated by altering the current optimal sequence during each step of the optimization in one of three ways: amino-acid substitution, addition or deletion. Here, addition was performed by adding any of the valid amino acids in a random position in one of the fragments. Deletions were performed by randomly deleting one amino acid from each fragment. Substitution was performed by randomly mutating a position within a chosen fragment in accordance with BLOSUM80 substitution frequencies. Two strategies were employed to improve convergence. First, we implemented energy landscape pavement (ELP) to counteract trapping in local minima based on a histogram of previously visited energy values [45]. Second, we used a temperature schedule derived by varying the acceptance probability *q* in the edge case where one of the generated candidates has a low energy and all the other candidates a high energy:

$$q=\frac{\exp(-E_{\min}/T)}{\left(n-1)\exp(-E_{\min}/T)+\exp(-E_{\min}/T\right)}$$ (1)

This acceptance probability was linearly varied between a value close to one and close to zero during the optimization, leading to an almost random acceptance in the beginning of the optimization and terminating by only allowing improvements to the solution in the final stage of optimization. The program source code together with the scripts to reproduce the conclusions and figures can be found at http://bis.ifc.unam.mx/software/dcf or https://github.com/cdiener/dcf (DOI: 10.5281/zenodo.30278).

### EMSA assays

1μg of pGREG546 plasmid was pre-incubated with the required quantity of each peptide to result in a 1:1 or 1:4 peptide charge to DNA charge ratio. Samples were incubated for 30 minutes and loaded on a 1% Agarose gel in SB buffer. DNA affinity was quantified by measuring the mean pixel intensity of the DNA bands with Fiji (http://fiji.sc) and normalizing to the intensity range between an empty spot on the gel and the intensity of the pGREG546 plasmid without any added peptide. Analysis scripts for the EMSA experiments as well as the raw data can be found with the distributed source code at http://bis.ifc.unam.mx/software/dcf.

### Minimal inhibitory concentrations for *antibacterial activity*

*Escherichia coli* strain DH5α was grown for 12 hours to mid-log phase and diluted to 8·10^5^ cells/ml by using the reference OD obtained by absorption spectroscopy. Each sample 100μL culture was treated with 50μL solution containing the respective peptide in the desired concentration or water in the case of the controls. Growth curves were measured on two 96 well half area plates with two replicates each followed for 24h using a plate reader (Synergy, USA). In order to minimize plate-specific effects due to variations in loading time or initial cell numbers, different plates measurements were normalized by the time-point where the water control samples reached their half-maximal OD before combining. The respective minimum from each growth curve was subtracted from the normalized data from individual or combined plates. Areas under the curves were obtained by applying a linear interpolation to the OD curves, which was then integrated exactly. Growth rates were obtained by log-transforming the growth curves, identifying the exponential phase by the linear parts in that log-transformation (OD_600_ between 0.05 and 0.5) and performing a linear regression in the exponential phase with the slope denoting the respective growth rate. All mentioned methodology was implemented in R (www.r-project.org) and is available as open source at http://github.com/cdiener/dcf.

Using the slope of the linear regressions determined in these experiments, we estimated the minimal inhibitory concentration (MIC) for *E*. *coli* of our 3 CPP designed peptides (α-NLS-C, NLS-α-CE and chimera) and tested it experimentally.

### Pheromone signaling assay

Pheromone signaling induction in *Saccharomyces cerevisiae* was measured by induction of eGFP fluorescence in a BY4741 strain containing the Fus1-eGFP construct in place of the wild type Fus1 (*MAT*a his3Δ1 leu2Δ0 met15Δ0 ura3Δ0 fus1::eGFP HIS3MX6, obtained from the yeast GFP collection) [33]. The BY4741 *fus1*::*eGFP* strain was grown overnight in YPD to mid-log phase and diluted to 10^6^ cells/mL. Samples were treated with the respective peptide concentrations and incubated for 3h before mixing 1:1 with low-diffusivity agarose suspended on a covered glass slide. Images were taken with an Olympus FV10 confocal microscope using a 60x objective with 2.0 optical aperture. Images were analyzed using Fiji (www.fiji.sc). The only modifications applied to the images were separation of the bright field and GFP channels, adjustment of brightness and contrast to 50% and addition of scale bars.

### Internalization assays in yeast

All 6 fluorogenic peptides (TAMRA-peptides, see Table 1) were prepared to 600μM in water as a 10X stock solution. Although all the peptides were apparently soluble in water, they all formed aggregates under the microscope. Thus, 3 cycles of sonications (sonicator Bransonic 32) for 3 min each were performed to dissolve these aggregates. BY4741 (*MAT*a) cells were grown overnight and diluted to reach an OD_600_ = 0.3. These cells were pelleted and suspended in water in a final volume of 1mL. 13.5μL of these cells were put on a 26.4x76.2x1.2 mm microscope slide (LAUKA) and 1.5μL of the stock solution for each peptide was added to a final volume of 15μL and covered with a 22x22x1.5 mm cover glass (Thomas scientific). These samples were observed under a multi-photon confocal microscope (Olympus FV1000) and pictures were recorded at 0, 10 and 20 min after the peptide addition. All images were taken as z-stacks with 10–14 z-levels. Bright field images were chosen from the in-focus z-stack, whereas TAMRA images denote the average fluorescence across the entire z-stack to obtain the fluorescence inside the entire cell volume. Images were analyzed using Fiji (http://fiji.sc/Downloads#Fiji).

### Internalization assays and nuclear localization assay in mammalian cells

Primary Mouse Embryo Fibroblasts (MEFs) were prepared from CD-1 mouse embryos of 17–18 days of gestation obtained from the animal house of the Instituto de Fisiología Celular, UNAM, following IACUC guidelines. MEFs were maintained in high glucose DMEM (10569, Gibco) containing 10% FBS (16000, Gibco), 5000 U penicillin/streptomycin (15140, Gibco) and plated at a density of 7x10^4^ cells/well in 12-well culture plate. MEFs were incubated at 37°C with 6 different peptides to a final concentration of 60μM. The peptides used were: α-NLS-C, α-NLS-C woRK, NLS-α-CE, NLS-α-CE woRK, chimera or chimera woRK (the woRK peptides are negative controls for internalization; see Table 1). Cells were incubated with the peptides for 20 min, then washed with PBS and fixed with 4% paraformaldehyde for 30 min at room temperature. Afterwards, samples were washed with PBS three times and then incubated with DAPI for 2 min at room temperature. Images were obtained by fluorescence microscopy (Nikon ECLIPSE Ti-U) using the NIS Elements, Basic Research software, Version 3.13.

### Circular dichroism

Circular dichroism spectra were recorded on a ChirascanTM CD Spectrometer (Applied Photophysics, UK) equipped with a peltier temperature controller. 0.3mg of each peptide was dissolved in 1mL of water or 50% aqueous trifluoroethanol and placed in quartz cuvettes of 0.1cm path length. Far-UV CD spectra were collected from 185 to 250 nm at 20°C. Spectra were averaged over 5 scans and the averaged blank spectra of solvents were subtracted. Ellipticity is reported as mean residue molar ellipticity and recorded in terms of molar elipticity [θ] (deg cm^2^ dmol^−1^). CONTIN/LL within the CDPro analysis software program [46] was used to estimate the secondary structure content of the peptides.

## Supporting Information

S1 Fig CPP probability distribution observed in PFAM families. (PDF)

S2 Fig *Escherichia coli* cell density measured in the presence of different peptides at different concentrations. (PDF)

S3 Fig Effect on growth rate of different peptides on *Escherichia coli* cells. (PDF)

S4 Fig Effect on growth rate at MIC for different peptides on *Escherichia coli* cells. (PDF)

S1 Table List of CPP probability values for every PFAM family. (CSV)
