# Supplementary figures and images for: Effective Design of Multifunctional Peptides by Combining Compatible Functions
Source: PLoS Comput Biol. 2016 Apr 20;12(4):e1004786. doi: 10.1371/journal.pcbi.1004786 (PMC4838304; doi:10.1371/journal.pcbi.1004786)

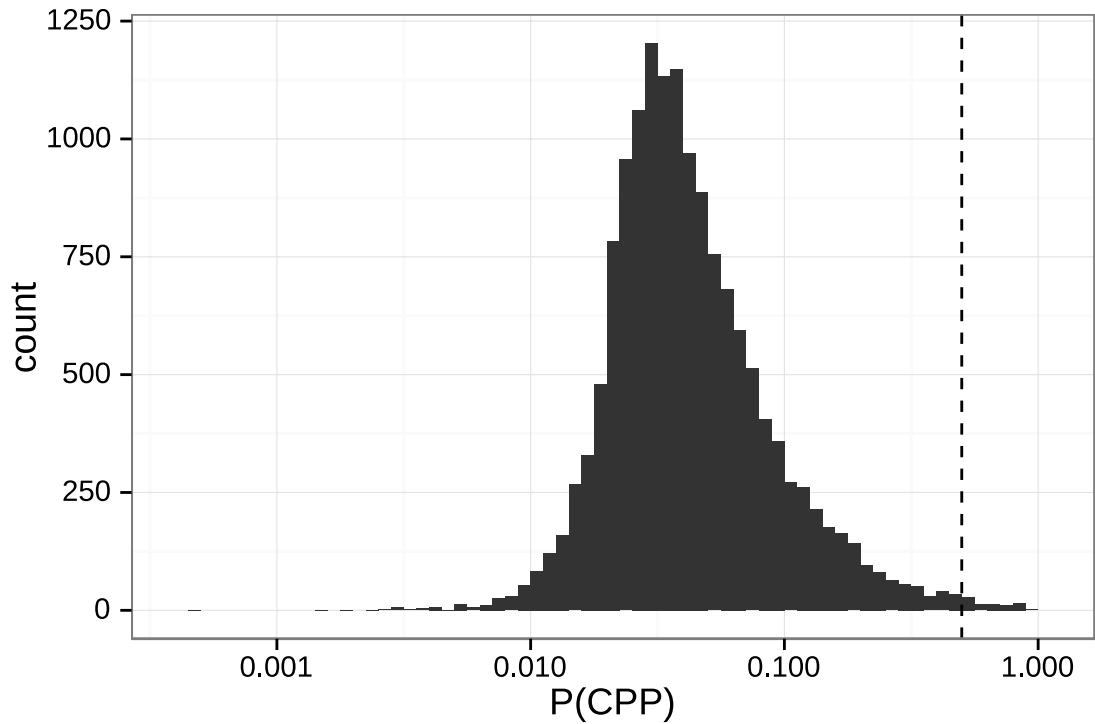

Supplement: S1 Fig — (PDF) [file pcbi.1004786.s001.pdf]

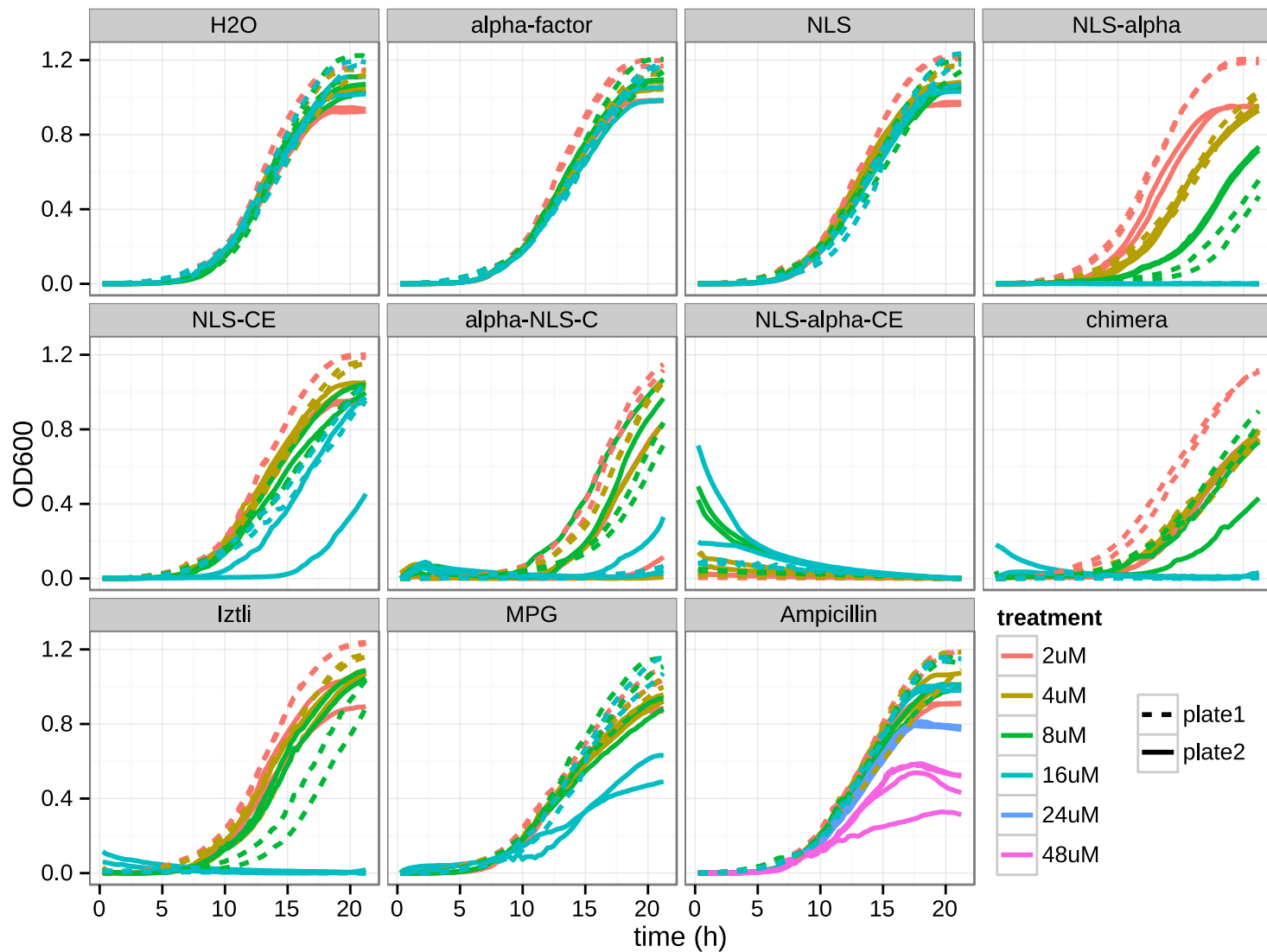

Supplement: S2 Fig — (PDF) [file pcbi.1004786.s002.pdf]

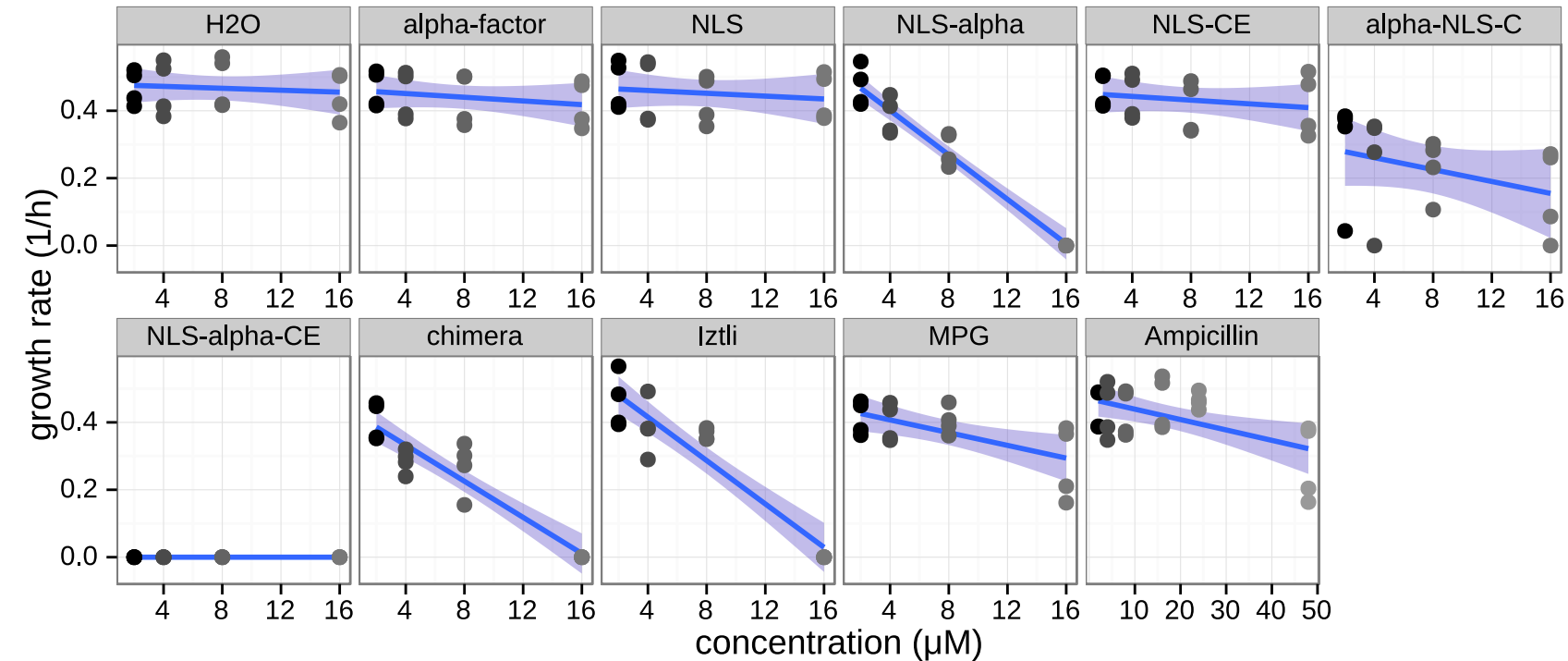

Supplement: S3 Fig — (PDF) [file pcbi.1004786.s003.pdf]

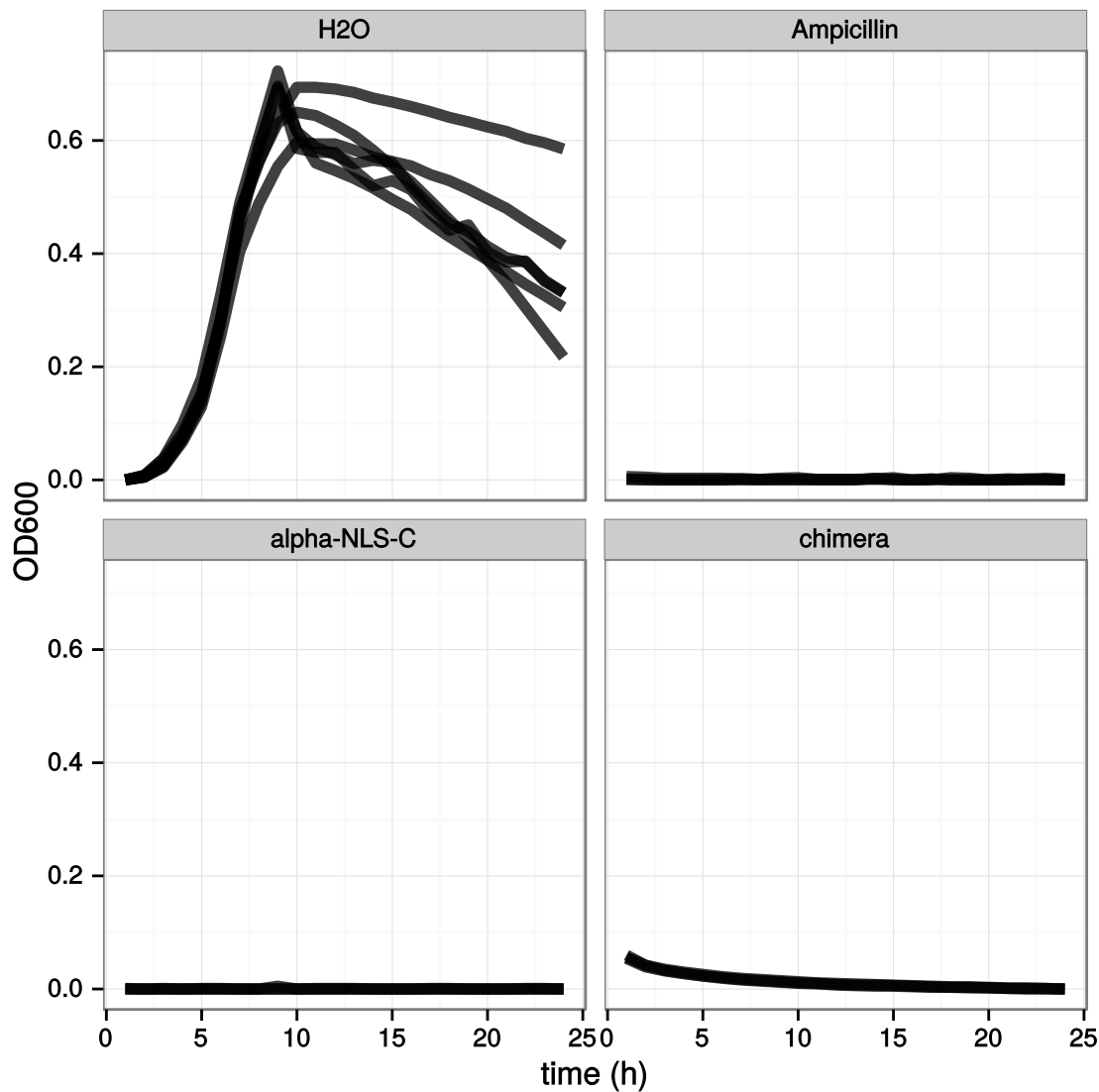

Supplement: S4 Fig — (PDF) [file pcbi.1004786.s004.pdf]
